# 2,4-Dichloro-6-({2-[(3,5-dichloro-2-hy­droxy­benzyl­idene)amino]­eth­yl}imino­meth­yl)phenol

**DOI:** 10.1107/S1600536812020235

**Published:** 2012-05-12

**Authors:** Ali Ourari, Lotfi Baameur, Sofiane Bouacida, Bouet Gilles, Allain Magali

**Affiliations:** a Laboratoire d’Electrochimie, d’Ingénierie Moléculaire et de Catalyse Redox (LEIMCR), Faculté des Sciences de l’Ingénieur, Université Farhat Abbas, Sétif 19000, Algeria; b Unité de Recherche de Chimie de l’Environnement et Moléculaire Structurale, CHEMS, Université Mentouri-Constantine, 25000 Algeria; c Laboratoire SONAS, E.A. 921, Faculte de Pharmacie, 16 Bd Daviers, 49045 ANGERS cedex 01, France; d MOLTECH Anjou UMR-CNRS 6200, 2 Bd Lavoisier, 49045 Angers cedex, France

## Abstract

The title mol­ecule, C_16_H_12_Cl_4_N_2_O_2_, lies about an inversion center. The symmetry-unique part of the mol­ecule contains an intra­molecular O—H⋯N hydrogen bond. In the crystal, mol­ecules are arranged in corrugated layers parallel to (-101). Weak π–π stacking inter­actions, with a centroid–centroid diatance of 3.7923 (13) Å, are present.

## Related literature

For the preparation of the title compound, see: Lu & Xia (2006 ▶); Trivedi *et al.* (1992 ▶). For the synthesis of similar compounds, see: Kadish *et al.* (1990 ▶); Taylor *et al.* (1991 ▶); Moutet & Ourari (1997 ▶) Ourari *et al.* (2008*b* ▶, 2011 ▶). For their applications, see: Ourari *et al.* (2008*a* ▶); Kadish *et al.* (1990 ▶).
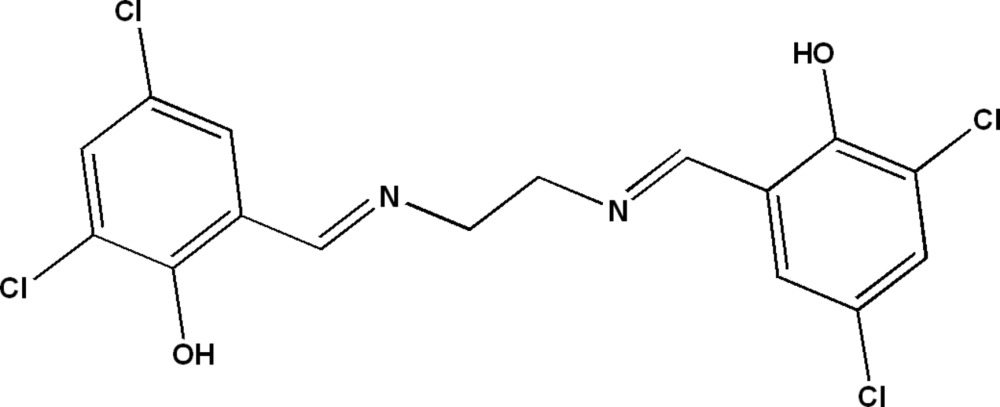


## Experimental

### 

#### Crystal data

C_16_H_12_Cl_4_N_2_O_2_
*M* *_r_* = 406.08
Monoclinic, 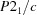

*a* = 7.529 (1) Å
*b* = 10.718 (2) Å
*c* = 10.759 (2) Å
β = 101.40 (2)°
*V* = 851.1 (3) Å^3^
*Z* = 2
Mo *K*α radiation
μ = 0.71 mm^−1^
*T* = 295 K
0.50 × 0.23 × 0.19 mm

#### Data collection

Stoe IPDS diffractometer
Absorption correction: gaussian (ABSGAUSS in *PLATON*; Spek, 2009 ▶) *T*_min_ = 0.794, *T*_max_ = 0.893
8137 measured reflections
1671 independent reflections
1192 reflections with *I* > 2σ(*I*)
*R*_int_ = 0.049

#### Refinement

*R*[*F* ^2^ > 2σ(*F* ^2^)] = 0.031
*wR*(*F* ^2^) = 0.081
*S* = 0.94
1671 reflections
110 parameters
H-atom parameters constrained
Δρ_max_ = 0.19 e Å^−3^
Δρ_min_ = −0.15 e Å^−3^

### 

Data collection: *EXPOSE* (Stoe & Cie, 1999 ▶); cell refinement: *SELECT* and *CELL* (Stoe & Cie, 1999 ▶); data reduction: *INTEGRATE* (Stoe & Cie, 1999 ▶); program(s) used to solve structure: *SIR2002* (Burla *et al.*, 2005 ▶); program(s) used to refine structure: *SHELXL97* (Sheldrick, 2008 ▶); molecular graphics: *ORTEP-3 for Windows* (Farrugia, 1997 ▶) and *DIAMOND* (Brandenburg & Berndt, 2001 ▶); software used to prepare material for publication: *WinGX* (Farrugia, 1999 ▶).

## Supplementary Material

Crystal structure: contains datablock(s) global, I. DOI: 10.1107/S1600536812020235/lh5465sup1.cif

Structure factors: contains datablock(s) I. DOI: 10.1107/S1600536812020235/lh5465Isup2.hkl

Supplementary material file. DOI: 10.1107/S1600536812020235/lh5465Isup3.cml

Additional supplementary materials:  crystallographic information; 3D view; checkCIF report

## Figures and Tables

**Table 1:** Hydrogen-bond geometry (Å, °)

*D*—H⋯*A*	*D*—H	H⋯*A*	*D*⋯*A*	*D*—H⋯*A*
O9—H9⋯N2	0.82	1.84	2.562 (2)	147

**Fig. 1.**
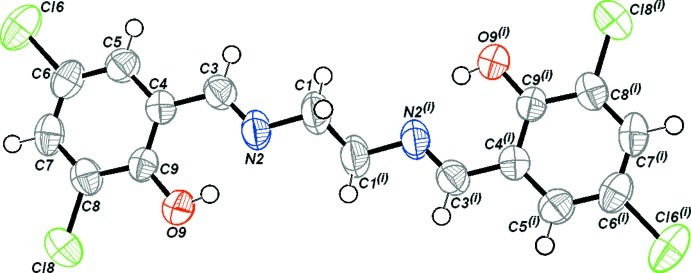
The molecular structure of the title compound with displacement ellipsoid drawn at the 50% probability level. H atoms are represented as small spheres of arbitrary radii. Symmetry code: (i)-x+1, -y, -z

**Fig. 2.**
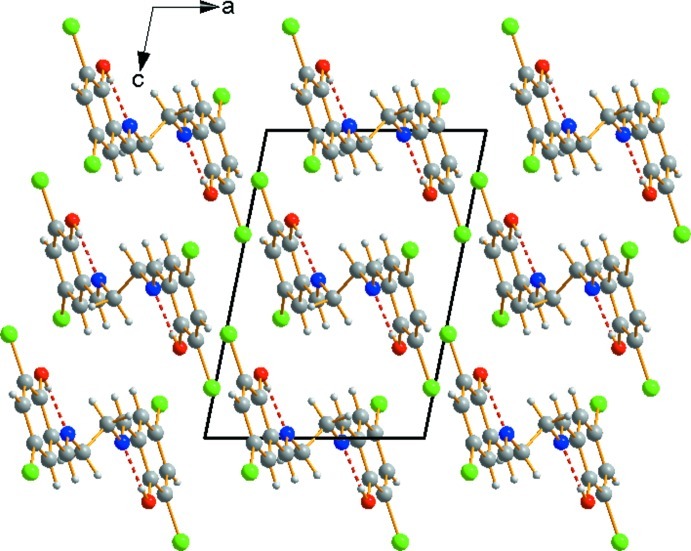
Layers of molecules paralel to (-101). Dash lines indicate hydrogen bonds.

**Fig. 3.**
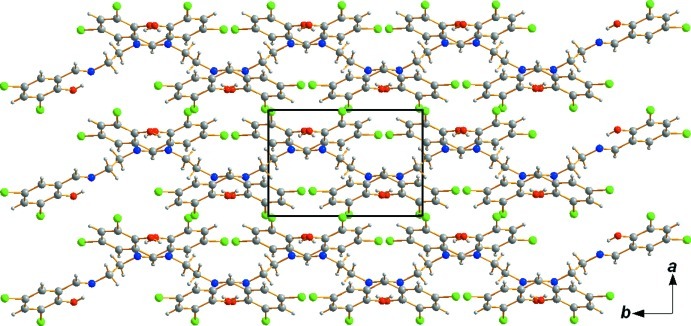
A projection of part of the crystal structure along [001].

## supplementary crystallographic information

### Comment

The synthesis of new chelating agents such as Schiff bases became an extensive area of research owing to their high structural versatility. This is due to their high ability to coordinate transition metals leading to the corresponding complexes. This class of compounds may be involved in many applications as in coordination chemistry, biology, analysis, catalysis and electrocatalysis (Ourari *et al.*, 2008*a*; Kadish *et al.*, 1990). Herein, we report the preparation and crystal structure the of the title compound. These type of polyhalogenated ligands are endowed with high resistance towards oxidation reactions seeing that the chlorine atoms are adequately grafted at *ortho* and *para*-positions of the phenolic entities, preventing their further oxidation reactions as it was early reported for the porphyrinic complexes (Kadish *et al.*, 1990; Taylor *et al.*, 1991; Moutet *et al.*, 1997). Some mononuclear complexes of Schiff base-Mn(III) compounds have been synthesized and used as catalysts towards epoxidation of olefins. This showed that the dihalogenated complexes behaved as the most efficient catalysts. Recently, we have as confirmed this observation when studying their analogues such as as those of iron(III) (Ourari *et al.*, 2008*b*) and ruthenium(III) (Ourari *et al.*, 2011) for the same oxidation reactions.

The molecular structure of (I) is shown in Fig. 1. The asymmetric unit of the title compound, consists of one-half of the molecule, with the other half generated by a crystallographic inversion centre. The crystal packing can be described as corrugated layers paralel to (-101) (Fig. 2). Fig. 3 shows the crystal structure with helical chains of molecules as a result of the 2_1_ screw axes. There are two intramolecular O—H···N hydrogen bonds in the molecule (Table 1, Fig. 2). Weak π–π stacking interactions with a centroid to centroid distance 3.7923 (13) Å are present between inversion related molecules.

### Experimental

All reagents were AR grade, obtained from Alfa Aesar Chemical Company. 3, 5-Dichlorosalicylaldehyde, 1,2-diaminoethane and anhydrous ethanol were used without any further purification. The ligand prepared in this work was performed according the literature (Lu *et al.*, 2006; Trivedi *et al.*, 1992). Thus, a solution of 3, 5-dichlorosalicylaldehyde 382 mg (2.10^-3^ mole) in anhydrous ethanol (10 ml) was dropwise added to a stirring ethanolic solution (10 ml), containing 60 mg (1.10^-3^ mole) of ethylenediamine. The reaction mixture was refluxed for about 2 h leading to the formation of a yellow precipitate. This precipitate was collected by filtration, washed several times with ethanol and then dried on phosphoric anhydride (P_2_O_5_), its yield is of 90%. The resulting compound (I) was re-crystallized from a solvent mixture dichloromethane/acetone with the volume proportions 90 and 10, respectively. Under the slow evaporation, suitable crystals for X-ray diffraction were obtained.

### Refinement

H atoms were located in difference Fourier maps but introduced in calculated positions and treated as riding on their parent atoms (C and O) with C—H = 0.93 Å (methine, aromatic), 0.97 Å (methylene) and O—H = 0.82 Å (hydroxyl) with *U*_iso_(H) = 1.2*U*_eq_(C) or *U*_iso_(H) = 1.5*U*_eq_(O).

### Crystal data

**Table:**

C_16_H_12_Cl_4_N_2_O_2_	*F*(000) = 412
*M**_r_* = 406.08	*D*_x_ = 1.585 Mg m^−^^3^
Monoclinic, *P*2_1_/*c*	Mo *K*α radiation, λ = 0.71073 Å
Hall symbol: -P 2ybc	Cell parameters from 4871 reflections
*a* = 7.529 (1) Å	θ = 2.7–25.9°
*b* = 10.718 (2) Å	µ = 0.71 mm^−^^1^
*c* = 10.759 (2) Å	*T* = 295 K
β = 101.40 (2)°	Prism, yellow
*V* = 851.1 (3) Å^3^	0.50 × 0.23 × 0.19 mm
*Z* = 2	

### Data collection

**Table:**

Stoe IPDS diffractometer	1192 reflections with *I* > 2σ(*I*)
Detector resolution: 6.66 pixels mm^-1^	*R*_int_ = 0.049
Oscillation Phi Incr 2.1 deg scans	θ_max_ = 26.1°, θ_min_ = 2.7°
Absorption correction: gaussian (ABSGAUSS in *PLATON*; Spek, 2009)	*h* = −9→9
*T*_min_ = 0.794, *T*_max_ = 0.893	*k* = −13→13
8137 measured reflections	*l* = −13→13
1671 independent reflections	

### Refinement

**Table:**

Refinement on *F*^2^	Primary atom site location: structure-invariant direct methods
Least-squares matrix: full	Secondary atom site location: difference Fourier map
*R*[*F*^2^ > 2σ(*F*^2^)] = 0.031	Hydrogen site location: inferred from neighbouring sites
*wR*(*F*^2^) = 0.081	H-atom parameters constrained
*S* = 0.94	*w* = 1/[σ^2^(*F*_o_^2^) + (0.0486*P*)^2^] where *P* = (*F*_o_^2^ + 2*F*_c_^2^)/3
1671 reflections	(Δ/σ)_max_ < 0.001
110 parameters	Δρ_max_ = 0.19 e Å^−^^3^
0 restraints	Δρ_min_ = −0.15 e Å^−^^3^

### Special details

**Table:**

Geometry. All e.s.d.'s (except the e.s.d. in the dihedral angle between two l.s. planes) are estimated using the full covariance matrix. The cell e.s.d.'s are taken into account individually in the estimation of e.s.d.'s in distances, angles and torsion angles; correlations between e.s.d.'s in cell parameters are only used when they are defined by crystal symmetry. An approximate (isotropic) treatment of cell e.s.d.'s is used for estimating e.s.d.'s involving l.s. planes.
Refinement. Refinement of *F*^2^ against ALL reflections. The weighted *R*-factor *wR* and goodness of fit *S* are based on *F*^2^, conventional *R*-factors *R* are based on *F*, with *F* set to zero for negative *F*^2^. The threshold expression of *F*^2^ > σ(*F*^2^) is used only for calculating *R*-factors(gt) *etc*. and is not relevant to the choice of reflections for refinement. *R*-factors based on *F*^2^ are statistically about twice as large as those based on *F*, and *R*- factors based on ALL data will be even larger.

### Fractional atomic coordinates and isotropic or equivalent isotropic displacement parameters (Å^2^)

**Table:**

	*x*	*y*	*z*	*U*_iso_*/*U*_eq_	
C1	0.4564 (3)	0.03468 (18)	0.0456 (2)	0.0533 (5)	
H1A	0.3651	−0.0174	0.0720	0.064*	
H1B	0.5464	0.0559	0.1203	0.064*	
C3	0.3844 (3)	0.24891 (17)	0.05017 (19)	0.0418 (4)	
H3	0.4478	0.2490	0.1336	0.050*	
C4	0.3008 (2)	0.36389 (16)	−0.00494 (17)	0.0358 (4)	
C5	0.3094 (2)	0.47168 (17)	0.06874 (19)	0.0411 (4)	
H5	0.3694	0.4703	0.1530	0.049*	
C6	0.2293 (3)	0.57999 (16)	0.0169 (2)	0.0427 (5)	
C7	0.1393 (3)	0.58432 (16)	−0.1083 (2)	0.0438 (5)	
H7	0.0854	0.6580	−0.1425	0.053*	
C8	0.1302 (2)	0.47829 (17)	−0.18167 (18)	0.0409 (4)	
C9	0.2103 (2)	0.36623 (15)	−0.13247 (18)	0.0362 (4)	
N2	0.3722 (2)	0.14858 (14)	−0.01354 (16)	0.0449 (4)	
O9	0.1968 (2)	0.26567 (12)	−0.20694 (13)	0.0479 (3)	
H9	0.2404	0.2052	−0.1650	0.072*	
Cl6	0.23717 (8)	0.71310 (5)	0.11055 (6)	0.0639 (2)	
Cl8	0.01342 (8)	0.47988 (5)	−0.33747 (5)	0.06174 (19)	

### Atomic displacement parameters (Å^2^)

**Table:**

	*U*^11^	*U*^22^	*U*^33^	*U*^12^	*U*^13^	*U*^23^
C1	0.0655 (14)	0.0390 (10)	0.0563 (14)	0.0175 (9)	0.0141 (10)	0.0124 (9)
C3	0.0446 (10)	0.0426 (10)	0.0388 (10)	0.0057 (8)	0.0094 (8)	0.0054 (8)
C4	0.0348 (9)	0.0336 (9)	0.0398 (10)	0.0016 (7)	0.0093 (8)	0.0025 (8)
C5	0.0406 (10)	0.0405 (10)	0.0424 (11)	−0.0021 (8)	0.0085 (8)	−0.0027 (8)
C6	0.0432 (10)	0.0316 (9)	0.0568 (13)	−0.0051 (8)	0.0183 (9)	−0.0053 (9)
C7	0.0449 (10)	0.0290 (9)	0.0607 (14)	0.0041 (8)	0.0181 (9)	0.0073 (9)
C8	0.0395 (10)	0.0387 (10)	0.0453 (11)	0.0026 (8)	0.0099 (8)	0.0082 (8)
C9	0.0390 (9)	0.0302 (8)	0.0405 (10)	0.0018 (7)	0.0112 (8)	0.0003 (8)
N2	0.0485 (9)	0.0355 (8)	0.0508 (10)	0.0108 (7)	0.0097 (7)	0.0072 (8)
O9	0.0621 (9)	0.0359 (7)	0.0433 (8)	0.0086 (6)	0.0050 (6)	−0.0026 (6)
Cl6	0.0764 (4)	0.0381 (3)	0.0824 (4)	−0.0077 (3)	0.0282 (3)	−0.0184 (3)
Cl8	0.0731 (4)	0.0593 (3)	0.0478 (3)	0.0151 (3)	−0.0003 (2)	0.0117 (3)

### Geometric parameters (Å, º)

**Table:**

C1—N2	1.462 (2)	C5—H5	0.9300
C1—C1^i^	1.484 (4)	C6—C7	1.383 (3)
C1—H1A	0.9700	C6—Cl6	1.7410 (19)
C1—H1B	0.9700	C7—C8	1.378 (3)
C3—N2	1.269 (2)	C7—H7	0.9300
C3—C4	1.456 (2)	C8—C9	1.400 (2)
C3—H3	0.9300	C8—Cl8	1.732 (2)
C4—C5	1.395 (2)	C9—O9	1.335 (2)
C4—C9	1.406 (3)	O9—H9	0.8200
C5—C6	1.375 (3)		
			
N2—C1—C1^i^	109.9 (2)	C5—C6—C7	120.98 (17)
N2—C1—H1A	109.7	C5—C6—Cl6	119.66 (16)
C1^i^—C1—H1A	109.7	C7—C6—Cl6	119.34 (14)
N2—C1—H1B	109.7	C8—C7—C6	119.31 (16)
C1^i^—C1—H1B	109.7	C8—C7—H7	120.3
H1A—C1—H1B	108.2	C6—C7—H7	120.3
N2—C3—C4	121.22 (18)	C7—C8—C9	121.46 (18)
N2—C3—H3	119.4	C7—C8—Cl8	120.31 (14)
C4—C3—H3	119.4	C9—C8—Cl8	118.21 (15)
C5—C4—C9	119.92 (16)	O9—C9—C8	119.32 (17)
C5—C4—C3	120.11 (17)	O9—C9—C4	122.41 (15)
C9—C4—C3	119.97 (16)	C8—C9—C4	118.26 (16)
C6—C5—C4	120.07 (18)	C3—N2—C1	119.58 (18)
C6—C5—H5	120.0	C9—O9—H9	109.5
C4—C5—H5	120.0		
			
N2—C3—C4—C5	177.24 (17)	C7—C8—C9—O9	179.49 (17)
N2—C3—C4—C9	−2.4 (3)	Cl8—C8—C9—O9	1.1 (2)
C9—C4—C5—C6	0.1 (3)	C7—C8—C9—C4	0.2 (3)
C3—C4—C5—C6	−179.52 (17)	Cl8—C8—C9—C4	−178.22 (13)
C4—C5—C6—C7	0.0 (3)	C5—C4—C9—O9	−179.50 (16)
C4—C5—C6—Cl6	178.76 (13)	C3—C4—C9—O9	0.2 (3)
C5—C6—C7—C8	−0.1 (3)	C5—C4—C9—C8	−0.2 (2)
Cl6—C6—C7—C8	−178.80 (14)	C3—C4—C9—C8	179.41 (16)
C6—C7—C8—C9	−0.1 (3)	C4—C3—N2—C1	−179.34 (17)
C6—C7—C8—Cl8	178.35 (14)	C1^i^—C1—N2—C3	−139.3 (3)

Symmetry code: (i) −*x*+1, −*y*, −*z*.

### Hydrogen-bond geometry (Å, º)

**Table:**

*D*—H···*A*	*D*—H	H···*A*	*D*···*A*	*D*—H···*A*
O9—H9···N2	0.82	1.84	2.562 (2)	147
